# Data on the effect of oral feeding of Arachidonic acid or Docosahexanoic acid on haematopoiesis in mice

**DOI:** 10.1016/j.dib.2017.08.009

**Published:** 2017-08-09

**Authors:** Kedar Limbkar, Ankita Dhenge, Dipesh D. Jadhav, Hirekodathakallu V. Thulasiram, Vaijayanti Kale, Lalita Limaye

**Affiliations:** a National Centre for Cell Science, NCCS Complex, Savitribai Phule Pune University Campus, Pune 411007, India; b Chemical Biology Unit, Division of Organic Chemistry, CSIR- National Chemical Laboratory, Dr. Homi Bhabha Road, Pune 411008, India; c CSIR-Institute of Genomics and Integrative Biology, Mall Road, New Delhi 110007, India

## Abstract

Stem cells have peculiar property to self-renew and differentiate. It is important to control their fate in safe and effective ways for their therapeutic use. The mediators of essential polyunsaturated fatty acids (PUFAs) namely Arachidonic acid (AA) and Docosahexanoic acid (DHA) are known to play a role in haematopoiesis via various metabolic pathways [1]. However the direct effect of purified AA or DHA on haematopoiesis has not been well investigated yet. We have reported that oral administration of PUFAs enhanced haematopoiesis in mice [2]. Signaling Leukocyte Antigen Molecule (SLAM) (CD48^−^CD150^+^) phenotype consists of pure population of haematopoietic stem cells (HSCs). Herein we observed higher percentage of SLAM (CD48^−^CD150^+^) phenotype in the bone marrow (BM) cells of mice fed with AA or DHA compared to PBS fed control mice. Data from engraftment study depicts that BM from AA/DHA-fed mice showed higher absolute number of donor cells in recipient mice compared to control. The enhanced hematopoiesis observed in AA/DHA-fed mice was returned to normal when the mice were kept on normal diet for six weeks (after ten days of oral feeding). We confirmed GCMS (Gas Chromatography-Mass Spectroscopy) retention times of AA and DHA by co-injecting fatty acid extract from AA or DHA fed mice with purified AA or DHA standards respectively. Representative flow cytometry profile of Lin^−^Sca-1^+^c-kit^+^(LSK) cells showed higher expression of CXCR4 protein and ligands of Wnt, Notch1 signaling in BM of AA/DHA-fed mice.

**Specifications Table**

**Table:** 

Subject area	*Biology*
More specific subject area	*Nutritional metabolism, Nutritional Biochemistry*
Type of data	*Graph, figure*
How data was acquired	*Flow cytometry, Gas chromatography-mass spectroscopy (GC-MS)*
Data format	*Analyzed data*
Experimental factors	*C57/BL6 mice were fed with AA/DHA daily for ten days and their bone marrow cells were subjected to various assays for haematopoiesis*
Experimental features	*Bone marrow cells of mice were stained with specific antibodies for HSCs were analyzed by immunofluorescence method, For engraftment study, bone marrow cells from AA/DHA-fed mice were infused in irradiated congenic recipient mice and donor population was analyzed.*
Data source location	*Stem cell laboratory, Lab #4, National Center for Cell Science,*
	*Savitribai Phule Pune University Campus, Pune, India*
Data accessibility	*Data is available with this article*

**Value of the data**

• This data set is of value to the researchers seeking effect of oral administration of AA or DHA on haematopoiesis (phenotypic as well as *in vivo* engraftment*)*.
• Data could facilitate analysis of GCMS (Gas Chromatography-Mass Spectroscopy) chromatograms of AA/DHA.
• Data shows that Wnt, Notch1 and CXCR4 signaling are probable molecular mechanisms being activated in enhanced haematopoiesis of AA/DHA-fed mice.

## 1 Data

**Fig. 1:**
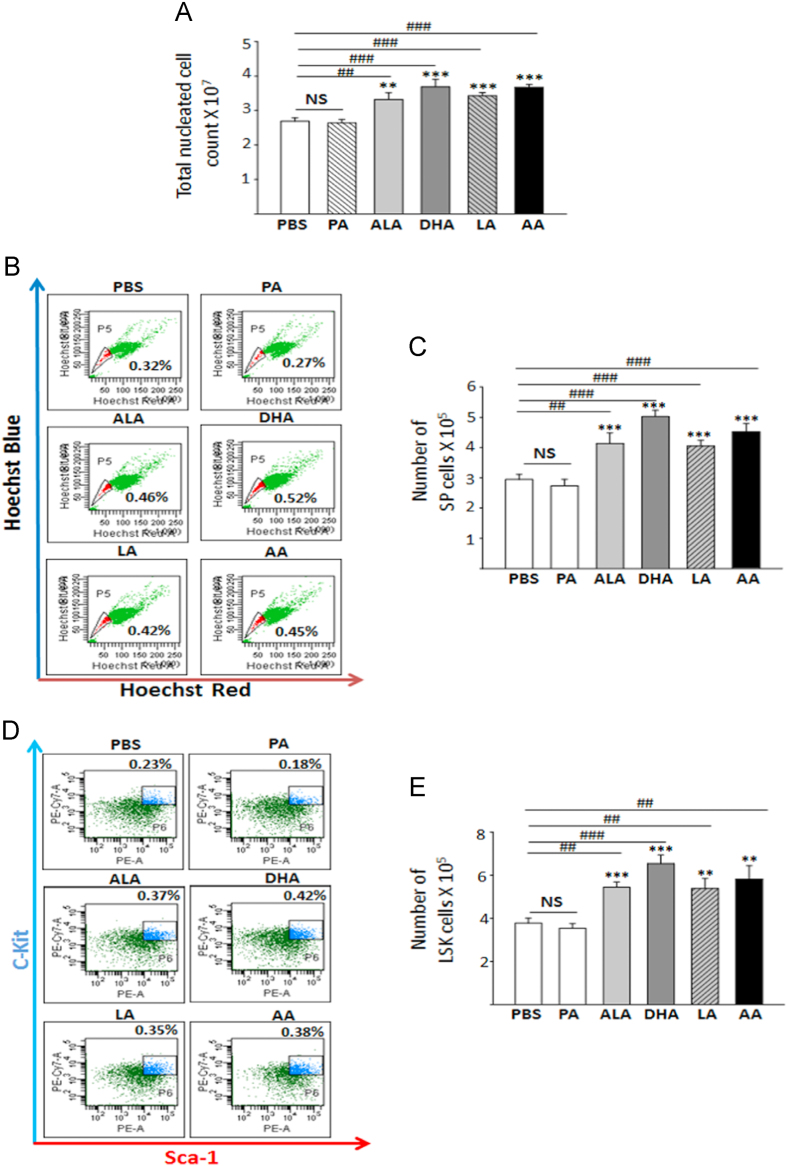
The data shown here gives comparative account of hematopoietic parameters of mice fed with PUFAs to control set-mice fed with Palmitic acid (SFA) or PBS. (A) Increase in TNC counts of PUFA-fed mice as compared to control mice. (B) Representative scatter plots of SP profile of BM cells of control sets and PUFA-fed mice. (C) Depicts absolute number of SP cells in BM of control and PUFA-fed mice. It is a cumulative data from six mice for each set. (D) Representative flow cytometry profiles for LSK cells of control and PUFA-fed mice. (E) Significantly increased LSK cells in AA/DHA-fed mice. ^*⁎*^Significance level when test sets were compared with Palmitic acid as control. # Significance level when test sets were compared with PBS as control.^*^p<0.05, ^**^p<0.01, ^***^p<0.001; n=6.

**Fig. 2:**
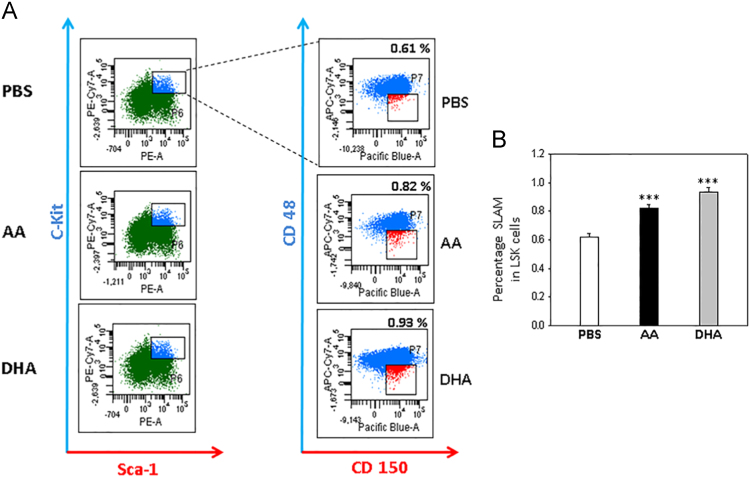
(A) Representative SLAM marker profiles of marrow cells of control and AA/DHA-fed mice in LSK gates. (B) Cumulative data of samples from six mice fed with PBS, AA or DHA. *p<0.05, **p<0.01, ***p<0.001; n=6.

**Fig. 3:**
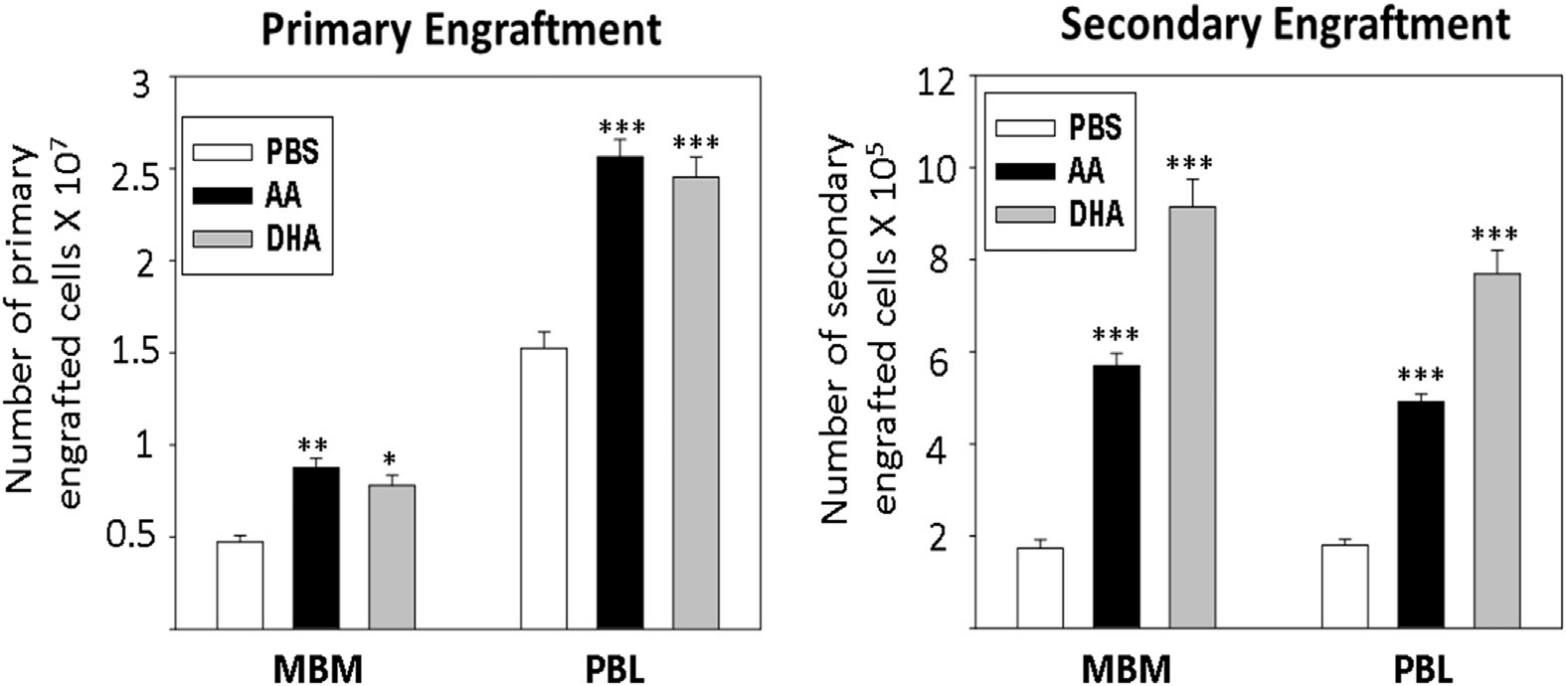
Left and right panels show absolute numbers of primary and secondary engrafted donor cells in recipient mice respectively when bone marrow cells of PBS/AA or DHA fed mice was infused in recipients. *p<0.05, **p<0.01, ***p<0.001; n=6.

**Fig. 4:**
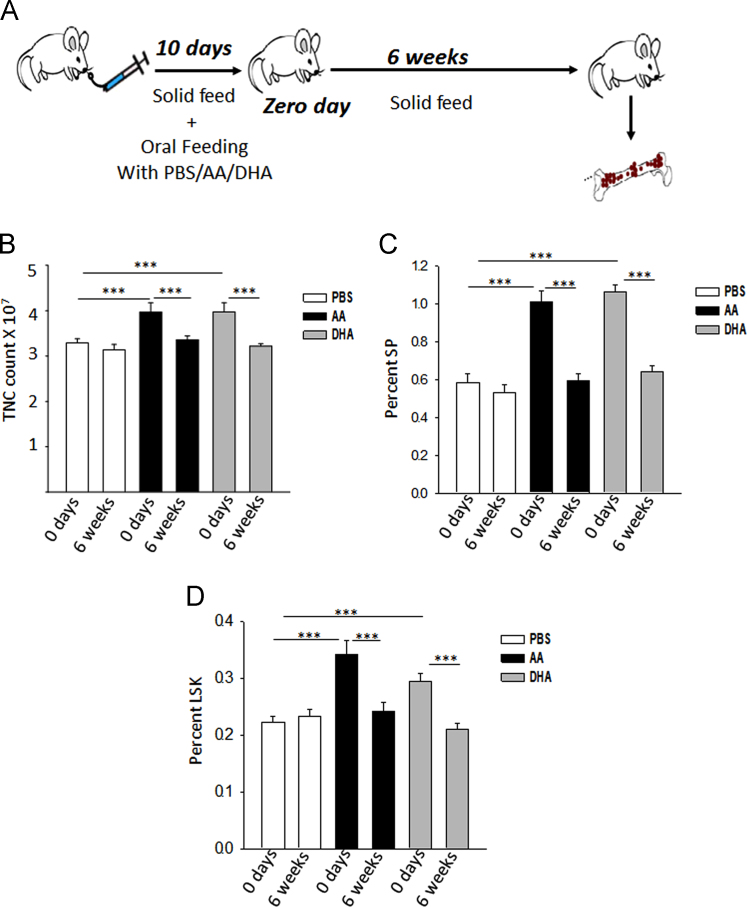
Zero day and 6 weeks indicates that mice were sacrificed immediately or after 6 weeks of oral feeding for 10 days respectively. (A) Flow diagram of this experiment.Increase in haematopoietic parameters observed on zero day, came back to normal after six weeks (B) TNC count (C) Percent SP (D) LSK percentage. *p<0.05, **p<0.01, ***p<0.001; n=6.

**Fig. 5:**
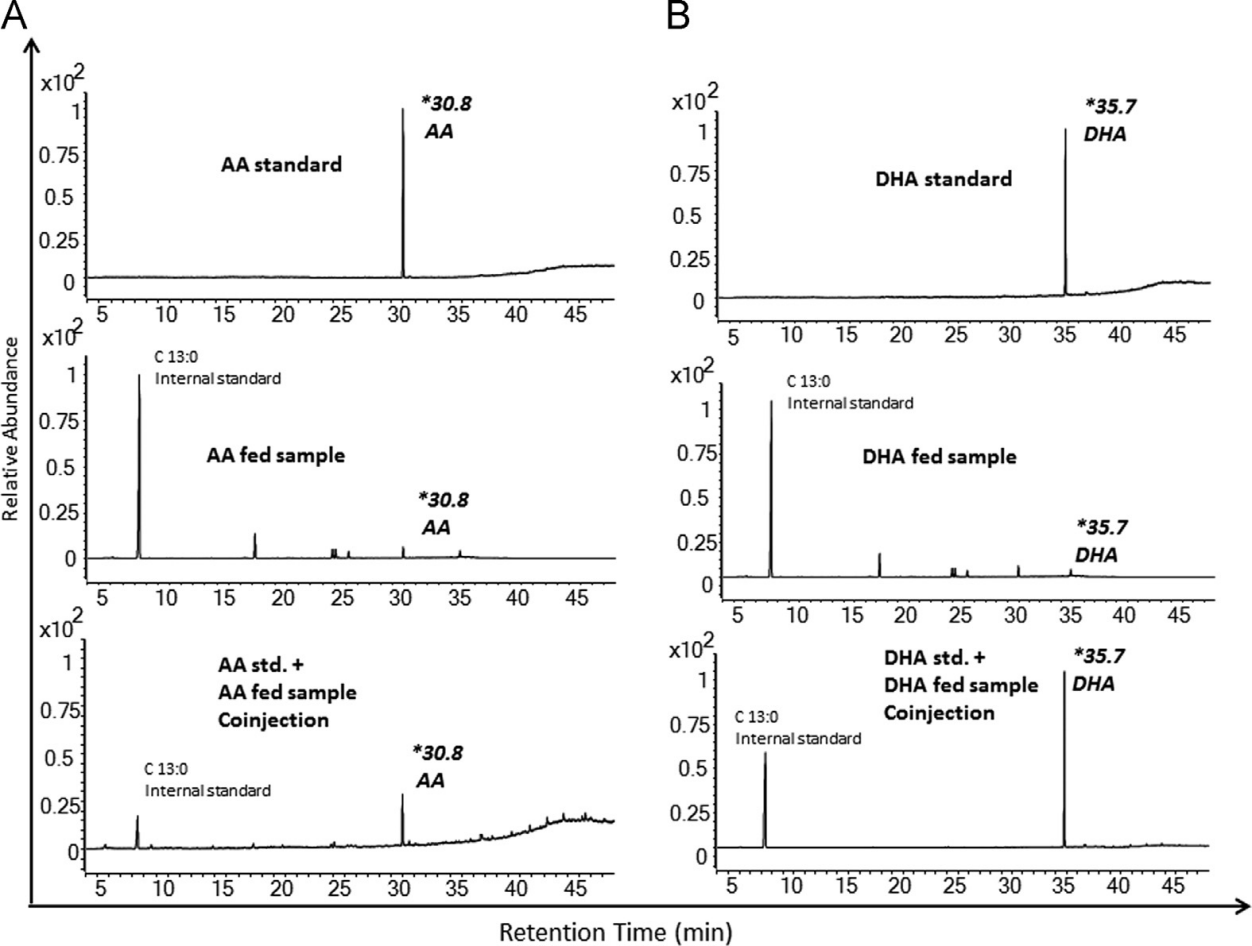
(A) GCMS chromatograms from top to bottom are of: purified AA methyl ester standard, Fatty acid methyl esters (FAMEs) of extract of BM cells of AA fed mice; Co-injection of FAME of purified AA standard and FAME extract of BM cells of AA fed mice. (B) Same panel for DHA. *p<0.05, **p<0.01, ***p<0.001; n=5.

**Fig. 6:**
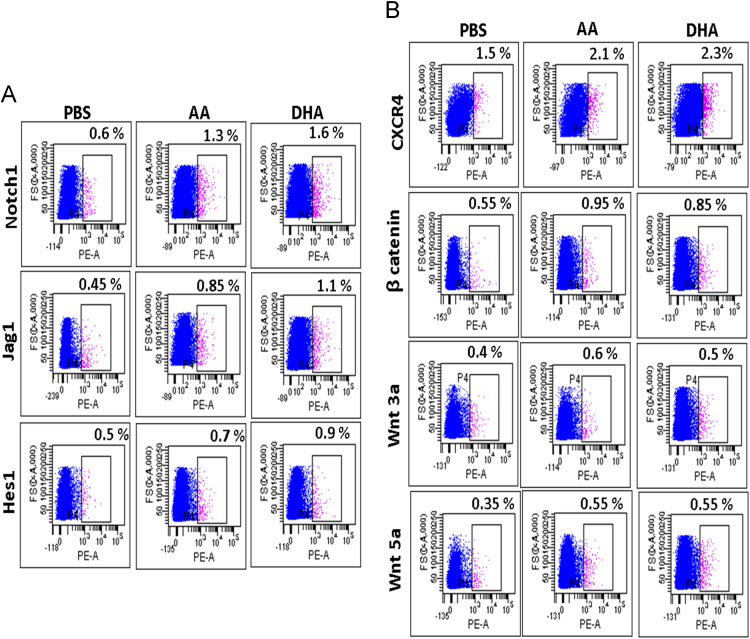
(A) Representative flow cytometry profiles of LSK cells of mice fed with PBS/AA/DHA stained with antibodies for Notch1, Jag1 and Hes1. Cells were first stained with LSK antibodies and the LSK population was analyzed. (B) for CXCR4, β catenin, Wnt3a and Wnt5a.

The data give comparative account of bone marrow (BM) cells of mice fed with AA/DHA with control mice fed *with* saturated fatty acid (SFA) – Palmitic Acid. Data of phenotypic flow cytometry analyses as well as statistics of bone marrow cells of mice fed with AA or DHA with control mice, is shown. The data also give graphical representation of various parameters for haematopoiesis of mice kept on normal diet after ten days feeding of AA or DHA(Fig.1 and Fig.2). Comparison of absolute numbers of engrafted donor cells from bone marrow of AA/DHA-fed mice is represented in the statistical format (Fig. 3).Comparative account between mice fed for ten days & sacrificed on next day and mice fed for ten days and sacrificed after six week, is shown (Fig. 4). The data of co-injection of fatty acid samples from bone marrow from AA or DHA fed mice with that of pure standards of AA and DHA is shown(Fig. 5). Data give representative flow cytometry profiles of bone marrow of AA/DHA-fed mice for ligands of Wnt and Notch1 signaling and CXCR4 protein(Fig. 6).

### 1.1 Oral feeding of mice

C57/BL6 (6–8 weeks) mice were fed daily through oral feeding gavage for ten days with 8 mg of either AA or DHA/mouse/day. PBS fed mice were kept as control. Mice fed with 8 mg of Palmitic acid were kept as additional control in initial experiments. Their BM cells were harvested and were subjected to assays for haematopoiesis. Detailed experimental design is described in Limbkar et al. [2].

### 1.2 SLAM marker expression analysis

LSK analysis was performed as per Limbkar et al. [2]. For SLAM (CD48^−^CD150^+^) antigen detection, LSK cells of PBS/AA/DHA fed mice were further stained with CD48APC-Cy7 and CD150 BV421. Cells were analyzed by flow cytometry.

### 1.3 Engraftment studies

This assay was done as described by Spangrude and coworkers, 1995. The detailed methodology is described in Limbkar et al. [2].

Absolute numbers of engrafted donor cells in recipient mice were calculated by the formula below.

Number of engrafted cells: Total nucleated cell count X Percentage engraftment100

### 1.4 Total nucleated cell count, Side population assay and LSK analysis

Total nucleated cell count, Side population assay and LSK analysis were performed as per Limbkar et al. [3].

### 1.5 Phenotypic marker expression

Bone marrow cells of PBS/AA/DHA fed mice were stained with monoclonal antibodies tagged with PE flurochrome - Notch1, Jag1, Hes1, CXCR4, β catenin, Wnt 3a and Wnt 5a (BD Bioscience, San Diego, USA) and incubated at 4 °C for 45 min. Cells were acquired on FACS Canto II (BD Bioscience, San Diego, USA). Data were analyzed using FACS Diva^™^ software. (BD Bioscience).

### 1.6 Co-injection experiment

Methyl esters of AA and DHA standards were co-injected with methyl esters from BM cells of AA and DHA fed mice and analyzed by GC–MS. Methyl esters were prepared as per Limbkar et al. [2]
